# HER-2 gene amplification in human breast cancer without concurrent HER-2 over-expression

**DOI:** 10.1186/2193-1801-2-386

**Published:** 2013-08-15

**Authors:** Shiuh-Wen Luoh, Betsy Ramsey, Amy Hanlon Newell, Megan Troxell, Zhi Hu, Koei Chin, Paul Spellman, Susan Olson, Edward Keenan

**Affiliations:** Division of Hematology and Medical Oncology, Oregon Health and Science University, 3181 SW Sam Jackson Park Road, MC L586, Portland, OR 97239 USA; Portland VA Medical Center, 3710 SW US Veterans Hospital Road, Portland, OR 97239 USA; Department of Physiology and Pharmacology, Oregon Health and Science University, 3181 SW Sam Jackson Park Road, Portland, OR 97239 USA; Department of Molecular and Medical Genetics, Oregon Health and Science University, 3181 SW Sam Jackson Park Road, Portland, OR 97239 USA; Department of Anatomic Pathology, Oregon Health and Science University, 3181 SW Sam Jackson Park Road, Portland, OR 97239 USA; OHSU Department of Biomedical Engineering and Center for Spatial Systems Biomedicine, Oregon Health and Science University, 3181 SW Sam Jackson Park Road, Portland, OR 97239 USA; Knight Cancer Institute, Oregon Health and Science University, 3181 SW Sam Jackson Park Road, Portland, OR 97239 USA

## Abstract

**Background:**

Testing for human epidermal growth factor receptor-2 (HER-2) in breast cancer is performed by either immunohistochemistry (IHC) or in situ hybridization (ISH). The growth factor receptor-bound protein-7 (GRB7) gene is in close proximity to HER-2 on chromosome 17q11-12 and codes a signal transduction molecule shown to be an independent adverse marker in breast cancer.

**Methods:**

HER-2 and GRB7 protein expression from 613 frozen breast tumors was determined by Western analysis. HER-2 protein results were confirmed with IHC. Commercial HER-2 FISH was performed on a subset of tumors with multi-probe FISH used to assess the extent of HER-2 gene amplification. mRNA expression was determined by Multi-plex RT-PCR.

**Results:**

Seven tumors with GRB7 protein over-expression scored HER-2 FISH amplified but had no HER-2 protein over-expression. Four of the 7 tumors showed elevated GRB7 but not HER-2 mRNA over-expression. The breast cancer cell line HCC3153 did not over-express HER-2 protein but showed HER-2 FISH amplification of a limited segment around the HER-2 gene. Ten breast cancer tumors from the TCGA database had gene copy number increases around HER-2 without HER-2 mRNA or protein over-expression.

**Conclusions:**

A subset of human breast cancers that test positive with FISH for HER-2 gene amplification do not over-express HER-2 protein. One mechanism for this discordance is the incomplete amplification of the smallest HER-2 region of chromosome 17q11-12, which includes GRB7. HER-2 gene amplification without protein over-expression is clinically significant because patients with such tumors are unlikely to benefit from HER-2 targeted therapy.

**Electronic supplementary material:**

The online version of this article (doi:10.1186/2193-1801-2-386) contains supplementary material, which is available to authorized users.

## Introduction

Amplification of chromosome 17q11-12 occurs in about 20-25% of breast tumors leading to over-expression of the human epidermal growth factor receptor 2 gene (HER-2 or ERBB2) (Slamon et al. [1987]; Slamon et al. [1989]; Ross et al. [2003]). The HER-2 gene encodes a tyrosine kinase receptor and is the best-studied gene present in the amplicon. Because chromosome 17q11-12 amplification was initially detected in frozen breast tumor specimens by Southern blot analysis using a HER-2 probe, it is historically known as HER-2 amplification (Tandon et al. [1989]; Kallioniemi et al. [1992]). Chromosome 17q11-12 amplification has been subsequently found to correlate with HER-2 over-expression on both the mRNA and protein levels in a molecularly fully characterized breast tumor cohort (Press et al. [2002]). Most studies of chromosome 17q11-12 amplification have focused on the HER-2 gene such that HER-2 gene amplification and HER-2 protein over-expression have come to be recognized as important markers of clinically aggressive breast cancer and the target of specifically directed therapies (Press et al. [2008]; Goldenberg [1999]; Xia et al. [2002]).

HER-2 protein, when over-expressed, is the molecular target for specific therapies such as Trastuzumab, a humanized monoclonal antibody that binds to the extracellular domain of the HER-2 protein (Goldenberg [1999]), and Lapatinib a small molecule inhibitor of the intracellular tyrosine kinase domain of both HER-2 and epidermal growth factor receptor (HER-1) (Xia et al. [2002]; Kim & Murren [2003]). Considerable data indicate that HER-2 protein over-expression is required for the responsiveness to either therapy (Press et al. [2008]; Mass et al. [2005]; Di Leo et al. [2008]). Both Trastuzumab and Lapatinib have received approval by the FDA for the treatment of HER-2 positive breast cancer and are associated with improved clinical outcome in metastatic (Slamon et al. [2001]; Geyer et al. [2006]) and, for Trastuzumab, early stage HER-2 positive breast cancer (Romond et al. [2005]; Piccart-Gebhart et al. [2005]).

Despite success in treating HER-2 positive breast cancer patients with these therapies, considerable debate continues to exist regarding which method of testing of HER-2 represents the best assessment of a patient’s HER-2 status (Bartlett et al. [2001]; Wolff et al. [2007]; Sauter et al. [2009]; Press et al. [2005]; Hammock et al. [2003]; Troxell et al. [2006]; Tse et al. [2011]; Pauletti et al. [1996]; Pauletti et al. [2000]; Perez et al. [2012]). The FDA has approved immunohistochemical (IHC) assay methods (Herceptest and Pathway), fluorescence *in situ* hybridization (FISH) assays (PathVysion; INFORM; and FISH pharmDx) and the newer chromogenic *in situ* hybridization (CISH) assays (SPOT-Light; INFORM dual CISH; and CISH pharmDx). The American Society of Clinical Oncology (ASCO) and the College of American Pathologists (CAP) recently created a set of joint guidelines for the laboratory evaluation of HER-2 status (Wolff et al. [2007]). They recommend either using IHC assays for initial evaluation of HER-2 status followed by reflex testing by FISH for some IHC categories (i.e. 2+) or utilization of FISH in initial testing (Wolff et al. [2007]).

In addition to HER-2, there are a number of other chromosome 17q11-12 genes, including closely neighboring GRB7, which may be amplified and over-expressed concurrently with HER-2 (Luoh [2002]; Kao & Pollack [2006]; Kauraniemi & Kallioniemi [2006]; Bai & Luoh [2008]; Stein et al. [1994]; Glynn et al. [2010]). The GRB7 gene codes for a multi-domain signal transduction molecule, and is known to play important roles in tumor growth and migration (Shen & Guan [2004]). The GRB7 protein can interact with HER-2 and multiple other signaling proteins, including receptor and non-receptor tyrosine kinases (Shen & Guan [2004]). Located less than 15 kb away from the HER-2 gene, the GRB7 gene is contained well within the smallest amplified region of the HER-2 amplicon on chromosome 17q11-12. Amplification of GRB7 and other neighboring genes is typically associated with increased transcriptional activation and may have significant prognostic and predictive value in breast cancer treatment independent of the HER-2 gene (Ramsey et al. [2011]; Nadler et al. [2010]).

Though they are usually over-expressed together, our Western blotting analysis of 563 breast tumors from a well-annotated breast tumor repository found a significant discordance in GRB7 and HER-2 protein over-expression, allowing us to investigate the independent prognostic significance of GRB7 protein over-expression in breast cancer (Ramsey et al. [2011]). We found that patients whose tumors had isolated GRB7 protein over-expression had the worst prognosis. In addition, our studies revealed that protein over-expression of GRB7 is a stronger independent adverse prognostic factor than HER-2 over-expression (Ramsey et al. [2011]), a conclusion which is seemingly at odds with the widely held perspective of the role of HER-2 protein over-expression as the sole driver of adverse prognosis in chromosome 17q11-12 amplified breast tumors. The role of GRB7 protein in breast cancer was recently further validated when GRB7 was found to be the only significant adverse prognostic factor from among 394 gene candidates examined in a uniformly treated patient population with triple negative breast cancer, an especially aggressive form of the disease (Sparano et al. [2011]; Giricz et al. [2011]).

With the current study we seek to elucidate possible mechanisms of isolated GRB7 over-expression and to explore resultant implications for current HER-2 testing and treatment regimens.

## Methods

### Tissues, cell culture, and databases

Tumor specimens analyzed in this study were derived from the Oregon Health and Science University (OHSU) Knight Cancer Institute Breast Cancer Repository (Ramsey et al. [2011]; Christianson et al. [1998]; Saez et al. [2006]). These tumors were originally submitted by community hospitals in Oregon, Washington and Alaska to the College of American Pathologists (CAP) - certified OHSU Hormone Receptor Laboratory between 1985-1998, for the purpose of routine evaluation of estrogen and progesterone receptor proteins. Tumors were snap-frozen within thirty minutes of biopsy or mastectomy. Frozen tissues remaining following the completion of the analysis were banked and have been maintained continuously frozen at –80 C. A waiver of informed consent and HIPAA authorization for research use of remaining tissue was approved by Institutional Review Boards (IRB) at the Oregon Health and Science University and community hospitals in accordance with federal and local privacy laws (OHSU IRB # IRB00000211).

Snap-frozen primary breast tumor tissues were also purchased from the National Disease Research Interchange (NDRI). Tumor pathology was verified and anonymized at the sites of origin and obtained according to protocols approved by local Institutional Review Boards.

Human breast cancer cell lines including HCC 3153 were obtained from ATCC and the laboratory of Dr. Joe Gray (OHSU Knight Cancer Institute) and maintained in RPMI with 10% FCS (Neve et al. [2006]). Protein extraction and Western blotting analysis for HER-2 and GRB7 protein expression were performed as previously described (Ramsey et al. [2011]).

The Cancer Genome Atlas (TCGA) project includes a database of comprehensive genomic and epigenetic information currently for 847 primary breast tumors. Data are deposited in standard formats in the TCGA Data Coordinating Center and are available on line through a data portal (Cancer Genome Atlas [2012]).

### HER-2 fluorescence in situ hybridization (FISH) analysis

HER-2 FISH was performed using the FDA-approved Abbott PathVysion kit on 4 μm frozen sections. Our protocol was adjusted from the FDA recommended protocol to fit within the confines of our study. This kit contains a probe for the HER-2 locus (17q12, SpectrumOrange) and the chromosome 17 centromere (CEP17) as the chromosome 17 control probe (D17Z1,17p11.1-q11.1, SpectrumGreen). OCT compound was removed by three 8-12 hour washes in 4°C 70% EtOH. Following removal of OCT compound, slides were fixed in 3:1 methanol:acetic acid for 5 minutes and baked at 95°C for 5 minutes. The preparations were then pepsin treated, denatured, hybridized, washed, and counterstained according to the manufacturer’s instructions. Fluorescence was visualized on a CytoVysion image capture system (Applied Imaging, San Jose, CA) with Nikon E800 (Nikon, Melville, NY) microscope. HER-2 and CEP17 signals were enumerated in 25 to 60 cells and a total HER-2/CEP17 ratio was calculated. HER-2 signal counts greater than 10 were considered too numerous to count and scored as >10, as defined by clinical laboratory standards. A ratio of >2.2 was considered to be amplified, <1.8, not amplified, and 1.8 to 2.2, equivocal, based on ASCO/CAP guidelines.

### Multiple probe FISH

We developed a multi-probe FISH analysis to examine the extent of HER-2 gene amplification using two flanking probes that define the centromeric and telemetric ends of the smallest amplified region of the HER-2 region; BAC probe 3014 and 3079 respectively. Clones were purchased from Invitrogen as GeneHogs (HS996) cultures in glycerol stocks and grown in LB broth with chloramphenicol. BACs were prepared using a Qiagen Midi kit according to protocol. Sequencing was done to confirm clone using primers T7 and Sp6. Purified BAC DNA from 3014 and 3079 were labeled with an Abbott Nick Translation kit. Purified 5′ probe #3014 was hybridized and labeled with Abbott SpectrumGreen (green fluorescence). Purified 3′probe #3079 was labeled with Abbott SpectrumOrange (orange fluorescence), and the commercially available chromosome 17 centromere probe with Abbott SpectrumAqua (aqua fluorescence). Coalescence of red and green fluorescence signals yield yellow signals indicating that sequences represented by both probes are amplified in one contiguous unit. FISH scores represent the ratio of BAC 3014 and BAC 3079 probes to the chromosome 17 centromeric region (D17Z1).

BAC Probe #3014 begins 35 kb upstream of the HER-2 gene promoter and extends further upstream encompassing the centromeric end of the smallest region of amplification (Staaf et al. [2010]). The sequence of one end of the BAC insert has been previously reported (aq154496). We sequenced both ends of its insert and verified that BAC #3014 does reside upstream of HER-2 and that the insert spans 72 kb.

BAC probe #3079 begins in the middle of GRB7 gene and extends further downstream and away from the HER-2 gene towards the telomere. Both ends of the BAC insert have been sequenced and verified as reported (AQ121537 and AQ 121760). BAC #3079 maps immediately downstream and 15 kb away from the 3′-end of the HER-2 transcription unit. #3079 represents the telemetric end of the smallest region of amplification. The size of insert of #3079 is 136 kb.

### HER-2 immunohistochemistry (IHC)

HER-2 IHC was performed by the College of American Pathologist (CAP) certified OHSU Clinical Immunohistochemistry Laboratory using an FDA approved kit with modifications for frozen tissues. Four mm frozen sections were acetone fixed, and stained on an FDA-approved Ventana XT instrument using anti-HER-2/neu 4B5 Rabbit Monoclonal Antibody (Pathway^TM^, Ventana, Tucson AZ) and a protocol optimized for frozen tissue. As slides were not formalin fixed, antigen retrieval was not necessary. Prediluted primary antibody was applied for 16 min at 37 degrees. Washing, secondary antibody incubation, and detection were performed using standard instrument protocols with Ultraview detection (Ventana).

### Multi-plex RT-PCR assay

RNA was isolated from frozen tumor slices using an Ambion RNAqueous kit. The ProtoScript MuLV Taq RTPCR kit was used for the detection of GRB7 and HER-2 mRNAs from a single first strand cDNA synthesis followed by PCR amplification with HER-2 or GRB7 specific primers and *β-*actin as internal control (Invitrogen), respectively. The primer concentrations for amplification of GRB7 was 2 μM, HER-2; 1 μM and β-actin; 3 μM. Thermal cycling was initiated at 94°C for 5 minutes, followed by 34 cycles of denaturation at 94°C for 30 seconds, annealing at 60°C for 30 seconds, and extension at 72°C for 2 minutes. Final extension was 70°C for 5 minutes. Detection was by agarose gel visualization. The sequence and product length of the oligonucleotide primer pairs used in this study are as follows: *β-actin,* forward primer 5′- AAG AGA GGC ATC CTC ACC CT-3′*,* reverse primer 5′- TAC ATG GCT GGG GTG TTG AA - 3′, product length 218 bp. GRB7 (growth factor receptor-bound protein 7) forward primer 5′ - AGG AAA CTT CGA GAG GAG GA- 3′, reverse primer 5′ - TTG GAC TCG TTC ACA TCT GC -3′, product size 847 bp. HER-2 (human epidermal growth factor receptor 2) forward primer 5′ - GGA AAC CTG GAA CTC ACC TA- 3′, reverse primer 5′ - TTG GTG TCT ATC AGT GTG AGA - 3′, product size 388 bp.

## Results

**Figure 1:**
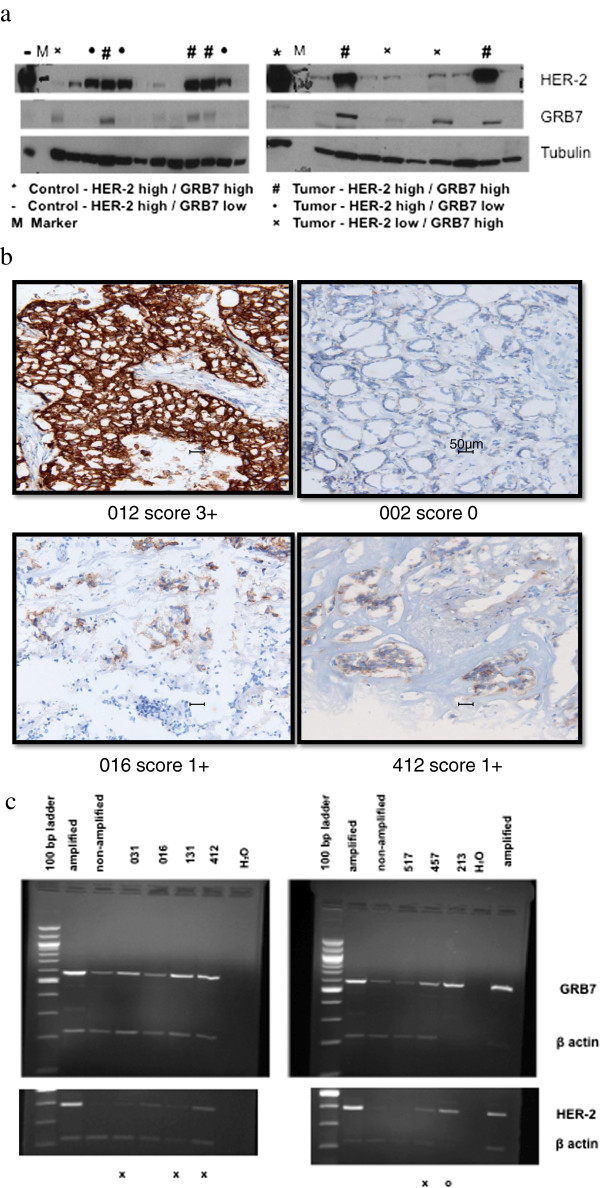
**GRB7 and HER-2 expression in frozen breast tumor tissues. a** GRB7 and HER-2 Western Blot Analysis. Densitometric analysis is presented in Additional file 1: Table S1. **b** HER-2 IHC testing of HER-2 FISH positive tumors which do not over-express HER-2 protein by Western blot analysis. Sample 012 is positive control with 3+ HER-2 over-expression; sample 002 is negative control with 0 HER-2 expression. Magnification bars represent 50 micron. **c** GRB7 and HER2 mRNA expression by multiplex RT-PCR. Top panel: GRB7 and β-actin, Bottom panel: HER-2 and β-actin. Amplified: positive control with HER-2 and GRB 7 over-expression. Non-amplified: negative control - without either HER-2 or GRB7 over-expression. H_2_O: negative control without RT product. X: 4 tumors over-expressing GRB7 but not HER-2. O: 1 tumor over-expressing both GRB7 and HER-2.

Previous results of Western analysis (Figure 1a and Additional file 1: Table S1) of primary breast cancer extracts from the OHSU Knight Cancer Institute Breast Cancer Tumor Repository for the presence of GRB7 and HER-2 proteins reported that GRB7 over-expression was found in the absence of HER-2 over-expression in 30 of 564 or 5% of tumors (Ramsey et al. [2011]). When tumors with this discordant GRB7/HER-2 protein expression pattern were analyzed for HER-2 gene amplification with an FDA approved, commercially available FISH assay (PathVysion, Abbott Labs), 6 of 27 samples scored amplified for HER-2 (Table 1). Additionally, one frozen tumor specimen among 50 obtained from National Disease Research Interchange (NDRI) was also found to have isolated GRB7 protein over-expression and scored HER-2 FISH amplified.

**Table 1:** **GRB7 and HER2 protein over-expression and HER2 gene amplification by FISH analysis**

Western result		Total (N)	HER2 FISH analyzed (N)	HER2 FISH amplified (N)	Amplified %
GRB7	HER2				
high	high	66	32	32	100
high	low	30	27	6	22
low	high	66	35	1	3
low	low	402	4	0	0

IHC analysis of the seven discordant tumors with a HER-2 immunohistochemistry (IHC) assay revealed scores of either 0 or 1+ for HER-2 protein expression. Some of these tumors exhibited a granular cytoplasmic pattern of HER-2 staining but the intensity and pattern were distinctly different from the 3+ strongly positive membrane staining pattern (Figure 1b) (Taylor et al. [1998]).

In order to examine the transcript levels of HER-2 and GRB7, we developed a multi-plex RT-PCR assay with *β-*actin as an internal control (Figure 1c). Among the 7 tumors, 4 showed elevated GRB7 mRNA expression but no elevated HER-2 mRNA expression. Two tumors demonstrated neither GRB7 nor HER-2 mRNA over-expression, while results for the seventh tumor indicated both HER-2 and GRB7 mRNA over-expression (Table 2). Sequencing of the coding region of HER-2 for the seventh tumor was conducted and revealed no apparent deleterious sequence changes in the coding region.

**Table 2:** **Summary of protein, mRNA and FISH results for HER-2 FISH positive tumors that do not over-express HER-2 protein and comparison group of HER-2 FISH positive tumors that do over-express HER-2 protein**

ID number	Western	Western	IHC	FISH	FISH	FISH	RT-PCR	RT-PCR
	GRB7	HER2	HER2	HER2	BAC 3079	BAC 3014	GRB7	HER2
**016**	high	low	1 +	2.58	NA	NA	low	low
**031**	high	low	Cyto Gran Stain	> 3.04	2.80	2.73	high	low
**131**	high	low	1 +	2.52	1.98	1.94	high	low
**412**	high	low	1 +	2.78	2.86	2.86	high	low
**457**	high	low	Cyto Gran Stain	5.21	5.27	5.32	high	low
**517**	high	low	Cyto Gran Stain	> 3.39	2.78	2.89	low	low
**213**	high	low	Cyto Gran Stain	4.44	2.48	3.59	high	high
**012**	high	high	3 +	> 5.13	NA	NA	high	high
**020**	high	high	3 +	5.28	NA	NA	NA	NA
**276**	high	high	3 +	4.90	NA	NA	NA	NA
**327**	high	high	3 +	4.48	3.77	3.77	high	high
**334**	high	high	3 +	4.93	NA	NA	high	high
**369**	high	high	3 +	4.98	4.06	4.02	high	high
**433**	high	high	NA	4.37	4.81	4.54	high	high
**772**	high	high	NA	4.59	5.18	5.18	high	high

FISH scores represent the ratio of signals derived from HER-2 region specific probes over that derived from the chromosome 17 centromeric region probe (D17Z1). NA = not analyzed.

**Figure 2:**
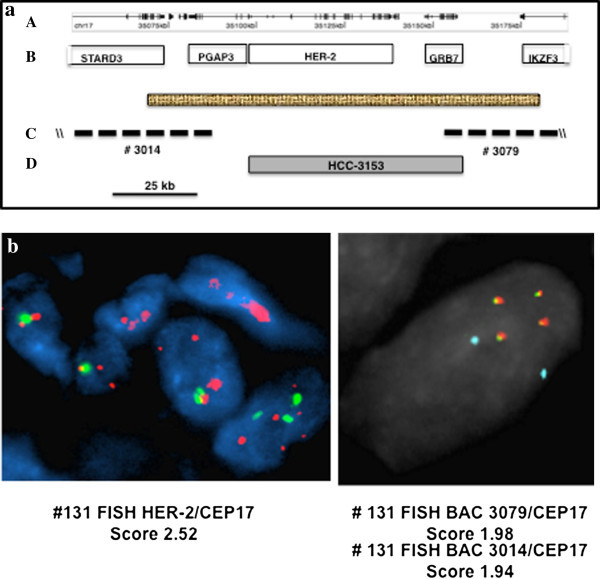
**A multiple-probe FISH analysis of the HER-2 and GRB7 segment from human chromosome 17q11-12. a A** 140 kb genomic segment centered on HER-2 from human chromosome 17q11-12. **a A** 140 kb genomic segment centered on HER-2 from human chromosome 17q11-12. **A**. The stippled box represents the smallest amplified region from HER-2 previously identified. **B**. Five genes from this region shown are STARD3, PGAP3, HER-2, GRB7 and IKZF3. **C**. The mapping positions of two BACs (#3014 and #3079) that define the centromeric and telomeric ends of the smallest amplified region are shown. **D**. Filled box represents the extent of gene amplification from the HER-2 region for the cell line HCC-3153. **b** FISH analysis of human breast tumor cells from tumor #031. HER-2 FISH was performed using the Abbott PathVysion kit on 4 μm frozen sections. Probe for the HER-2 locus (17q12, SpectrumOrange) and the chromosome 17 centromere (CEP17) as the chromosome 17 control probe (D17Z1,17p11.1-q11.1, SpectrumGreen). BAC 3079, BAC 3014. Purified 5′probe #3014 was labeled with Abbott SpectrumGreen (green fluorescence). Purified 3′probe #3079 was labeled with Abbott SpectrumOrange (orange fluorescence), and the commercially available chromosome 17 centromere probe with Abbott SpectrumAqua (aqua fluorescence). Coalescence of orange and green fluorescence signals yield yellow signals indicating that sequences represented by both probes are amplified in one contiguous unit.

Multi-probe FISH analysis, designed to examine the extent of HER-2 gene amplification in samples with apparent HER-2 gene amplification but no concurrent HER-2 protein over-expression, used two flanking probes that define the centromeric and telemetric ends of the smallest amplified segment of the HER-2 region; BAC probe 3014 and 3079 respectively (Figure 2a) (Staaf et al. [2010]). One sample out of 7 demonstrated decreased levels of gene amplification with these flanking probes; a ratio drop from 2.5 to below 2.0, indicating a lack of complete amplification of the smallest amplified region from HER-2 in this sample (Sample ID 131) (Figure 2b).

**Figure 3:**
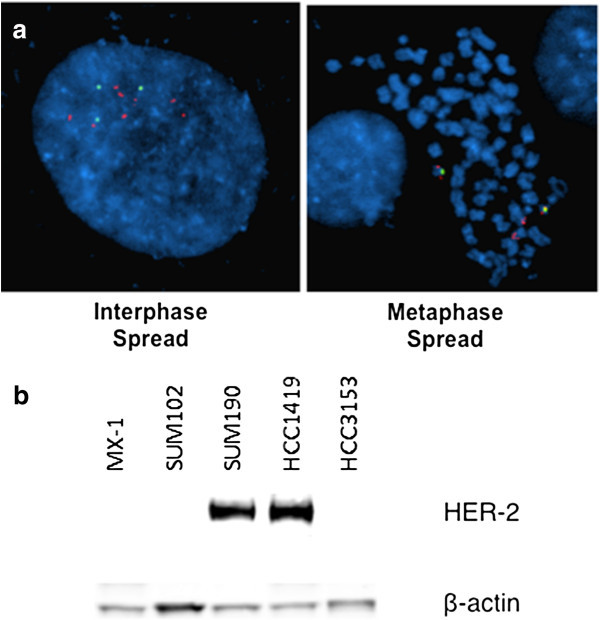
**A human breast cancer cell line with HER-2 gene amplification but no concurrent HER-2 over-expression. a** HER-2 gene amplification in breast cancer cell line HCC3153 Red: HER-2 by PathVysion probe; Green: Chromosome 17centromere. **b** Western analysis of HER-2 protein expression of breast cancer cell lines.

To confirm our observation that at least in some breast cancers apparent HER-2 gene amplification without HER-2 over-expression is due to incomplete, or unproductive, amplification of the smallest amplified HER-2 region, we examined an independent set of primary breast tumor data from The Cancer Genome Atlas Project (TCGA) and a panel of established breast cancer cell lines from SU2C (Stand up to Cancer) (Heiser et al. [2012]). The HCC 3153 cell line, which does not over-express HER-2 or GRB7 RNA, scored amplified by currently available HER-2 FISH testing with a ratio of 2.65 (Figure 3a). Array hybridization revealed that cell line HCC3153 has both HER-2 and GRB7 genes amplified but that the extent of the amplification does not extend to cover the smallest amplified region in its entirety (accession no. EGAS00000000059 in the European Genome-Phenome Archive (EGA); and accession no. E-MTAB-181 in the ArrayExpress) (44). Western analysis confirmed the lack of HER-2 protein over-expression (Figure 3b). HCC3153, therefore, is a cell line model of HER-2 gene amplification without HER-2 over-expression. A survey of gene copy number for 847 primary breast tumors available in the TCGA database found 10 tumors which had gene copy number increases in only part of the 140 kb genomic segment centering around HER-2. These samples have a log2 ratio of 0.5 or higher, a value commonly used as the cut-off for amplification in array analysis, for part of this region from the GISTIC2 database. Five samples out of ten had ratios greater than 1.0, a cut off for high level of 17q11-12 amplification. None of the samples demonstrated HER-2 mRNA over-expression based on RNAseq analysis in TCGA. Data regarding HER-2 protein expression determined by IHC, available for all but one tumor, showed that all specimens scored less than 3+ for HER-2 staining (Table 3).

**Table 3:** **TCGA tumors positive for partial HER-2 region amplification but with low HER-2 mRNA expression**

Tumor ID	GISICC2 values highest over Amplicon-140 kb	RNAseqV1 HER-2 values	HER-2 IHC
TCGA-D8-A1JG	0.58	1.09	2 +
TCGA-AN-A041	0.64	0.88	2 +
TCGA-A8-A070	0.72	- 5.50	1 +
TCGA-A8-A0AD	0.74	- 0.65	1 +
TCGA-D8-2A7G	0.78	-	2 +
TCGA-A2-A0YE	1.12	- 4.00	0 +
TCGA-C8-A26X	1.14	- 2.74	1 +
TCGA-A7-A13D	1.68	- 2.57	2 +
TCGA-A8-A06U	1.81	- 1.70	2 +
TCGA-BH-A1EN	6.00	- 3.90	-

## Discussion

Working with a well-annotated frozen breast tumor repository, our laboratory has been studying tumors with amplification of the chromosome 17q11-12 region. As part of these studies, we previously performed Western analysis of the expression of HER-2 and GRB7 protein, the products of two closely linked genes, which are often over-expressed in tandem when 17q11-12 amplification is present. Analysis of these data finds discordance in HER-2 and GRB7 protein over-expression and shows GRB7 protein over-expression to be a stronger independent predictor of adverse prognosis than the over-expression of the HER-2 protein. Additionally, we found that the breast cancer patient population with isolated GRB7 protein over-expression appears to have the worst prognosis (Ramsey et al. [2011]). Studies by others likewise report that GRB7 protein over-expression is an adverse prognostic factor in triple negative breast cancer (Nadler et al. [2010]; Sparano et al. [2011]).

In order to elucidate possible mechanisms involving GRB7 protein over-expression without HER-2 over-expression, we performed a standard clinical FISH analysis for HER-2 gene amplification. Surprisingly, 6 of 27 tumors scored positive for HER-2 gene amplification with this assay. This result was unexpected since these samples did not over-express HER-2 protein by Western analysis. Though these patients were diagnosed as HER-2 positive by FISH analysis, the gold standard for HER-2 clinical laboratory testing, such patients seem unlikely to benefit from HER-2 targeted therapy given the absence of HER-2 protein.

Because the accurate determination of HER-2 status is essential to treatment, we subjected our set of tumors to a standard clinical assay for HER-2 protein by IHC and found those results to be consistent with the lack of HER-2 protein over-expression found with Western analysis. However, using a Multi-plex RT-PCR assay, we found mixed results for HER-2 and GRB7 mRNA expression. Four out of 7 specimens over-expressed GRB7 but not HER-2 mRNA. This result seems consistent with over-expression of the GRB7 protein rather than HER-2 in some tumors. This suggests that GRB7 can be the focus for gene amplification and selective retention of the 17q11-12 segment and lends further evidence to the role of GRB7 protein over-expression as an independent adverse prognostic factor in breast cancer.

Two of 7 samples failed to over-express GRB7 or HER-2 mRNA despite isolated GRB7 protein over-expression. One of the 7 samples (sample ID 213) over-expressed both HER-2 and GRB7 mRNA but sequencing of the coding region of HER-2 mRNA for this tumor found no mutation in its open reading frame, suggesting post-transcriptional regulation of HER-2 expression. The mRNA and protein expression pattern in this tumor is nonetheless consistent with the concept of GRB7 gene being the focus of 17q11-12 amplification and selective retention.

Cases of HER-2 FISH positive but IHC negative tumors have been reported in the past, making up about 2% of all breast cancer patients or 10% of HER-2 FISH positive breast tumors (Sauter et al. [2009]; Lipton et al. [2010]). Much of this discrepancy has been attributed to pre-analytic and analytic issues associated with IHC of paraffin preserved sections. Our finding of frozen breast tumor samples that score positive for HER-2 gene amplification by FISH but do not over-express HER-2 protein by Western analysis nor HER-2 mRNA by RT-PCR, indicates some of these tumors may indeed be true HER-2 FISH false positives- i.e. HER-2 gene amplification without concomitant protein over-expression. Of note, we did not perform HER-2 FISH analysis of all 563 patient samples in our cohort, but based on the distribution and incidence of HER-2 FISH amplified cases in subsets of this tumor cohort we estimate 10% of tumors that were FISH amplified would not over-express HER-2 protein on Western analysis.

In an attempt to understand the mechanism of isolated GRB7 protein over-expression, we speculated that perhaps only a portion of the HER-2 gene is amplified. The HER-2 FISH probe utilized in the commonly employed Abbott PathVysion Kit covers a 190 kb genomic segment of chromosome 17q11-12 that contains both HER-2 and GRB7 genes and may score positive when only part of the HER-2 gene or GRB7 gene alone is amplified. Therefore we developed a multi-probe FISH assay with a set of flanking probes that demarcate the centromeric and telomeric ends of the smallest amplified region, which was previously identified as 90 kb in length (Staaf et al. [2010]). Utilizing this strategy, we were able to perform multi-probe FISH analysis on 6 of the 7 samples. Four of the samples (131, 517, 213 and 031) showed different and lower copy numbers when flanking probes were used rather than the commercial probe, which targets the central portion of the amplified region. In the case of specimen 131, the amplification appears to fall short of covering the smallest amplified region in its entirety, possibly explaining why this sample, though scoring positive for HER-2 gene amplification by current commercial assay, fails to over-express HER-2 protein or HER-2 mRNA. Current HER-2 FISH testing strategy cannot accurately discern the HER-2 amplification status of this sample.

We turned to the SU2C and TCGA databases to search for a similar pattern of HER-2 protein expression and gene amplification in breast cancer cell lines and other patient cohorts. We found that the breast cancer cell line HCC 3153 displays an amplification in only part of the smallest amplified region from the 17q11-12 region based on array analysis, though scoring positive with the standard HER-2 FISH assay. This cell line fails to over-express either HER-2 or GRB7 mRNA or protein. Thus we have identified a cell line model of HER-2 gene amplification without protein or mRNA over-expression that independently confirms our observation using banked frozen tumors.

A survey of the TCGA database (N = 847) found 10 tumors with only partial amplification of the HER-2 region based on array hybridization, though these tumors would be expected to score amplified with currently available HER-2 FISH assays. None of these tumors exhibited HER-2 mRNA or protein over-expression. This result indicates in this cohort derived from the TCGA database that about 5% of HER-2 amplification events occur without concurrent HER-2 over-expression and that this is due to partial amplification of the HER-2 region. This observation helps confirm our view that incomplete or partial amplification of the smallest amplified region of HER-2 may be problematic for current FISH and CISH assay methods that use only a single HER-2 probe.

In conclusion, our work provides evidence that the GRB7 gene may be the focus of some cases of the chromosome 17q11-12 amplification and selective retention, further strengthening the prognostic importance of GRB7 protein over-expression in breast cancer. In addition, we have established that a subset (approximately 5-10%) of primary breast cancers, considered as HER-2 amplified by the current FISH assay, are actually HER-2 false positive due to incomplete amplification of the smallest HER-2 region. Given this circumstance, multi-probe FISH may represent a potential strategy to avoid this pitfall and improve HER-2 FISH testing accuracy. Accurate determination of HER-2 status is essential since patients whose tumors have HER-2 gene amplification but no protein over-expression are unlikely to benefit from current HER-2 targeted therapies.

## Electronic supplementary material

Additional file 1: **Densitometric analysis of Western blotting results of Figure** 1 **a.** (TIFF 1 MB)
