# Supplementary figures and images for: HER-2 gene amplification in human breast cancer without concurrent HER-2 over-expression
Source: Springerplus. 2013 Aug 15;2:386. doi: 10.1186/2193-1801-2-386 (PMC3791222; doi:10.1186/2193-1801-2-386)

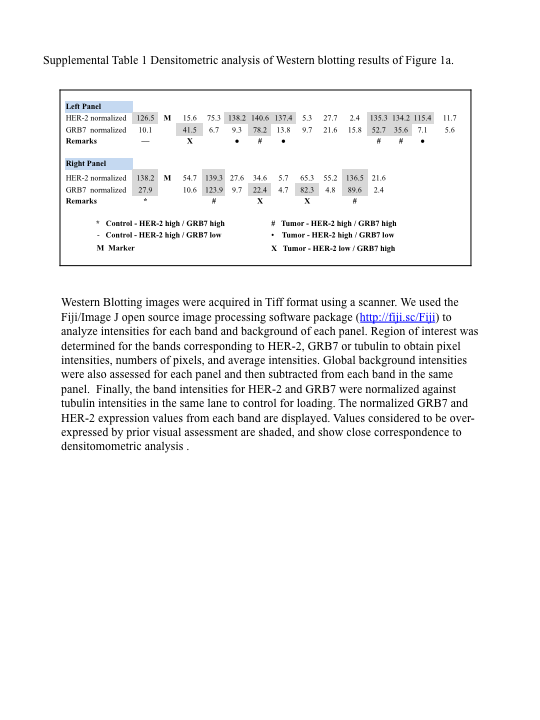

Supplement: Supplementary file 1 — Additional file 1: Densitometric analysis of Western blotting results of Figure 1 a. (TIFF 1 MB) [file 40064_2013_544_MOESM1_ESM.tiff]
